# The Role of Polymorphisms in Vitamin D-Related Genes in Response to Vitamin D Supplementation

**DOI:** 10.3390/nu12092608

**Published:** 2020-08-27

**Authors:** Sara Tomei, Parul Singh, Rebecca Mathew, Valentina Mattei, Mathieu Garand, Mariam Alwakeel, Elham Sharif, Souhaila Al Khodor

**Affiliations:** 1 Research Department, Sidra Medicine, Doha 26999, Qatar; psingh@sidra.org (P.S.); rmathew1@sidra.org (R.M.); valentinamattei3@gmail.com (V.M.); mathieu.garand@gmail.com (M.G.); 2 Department of Biomedical Sciences, College of Health Sciences, Qatar University, Doha 26999, Qatar; mariamalwakeel94@gmail.com

**Keywords:** vitamin D, polymorphisms, single-nucleotide polymorphism, SNP, 25-hydroxyvitamin D, 25(OH)D, vitamin D deficiency, blood

## Abstract

Background. Vitamin D deficiency represents a major healthcare problem. Vitamin D status is influenced by genetic and environmental determinants. Several observational studies have evaluated the association of single-nucleotide polymorphisms (SNPs) in vitamin D-related genes and vitamin D levels. Nevertheless, little is known about the role of these SNPs in the response to vitamin D supplementation. We conducted an interventional study to define the association between SNPs in vitamin D-related genes and the response to vitamin D supplementation in 100 self-reported healthy women of Arab ancestry for the majority. Methods. A total of 100 healthy female subjects received a weekly oral dose of 50,000 IU vitamin D for 12 weeks. Serum vitamin D concentration and metabolic profiles were measured at baseline and 12 weeks post-vitamin D supplementation. The genotypes of 37 SNPs selected from previously reported vitamin D-related genes have been assessed by Fluidigm genotyping assay. Results. Rs731236 (VDR gene) and rs7116978 (CYP2R1 gene) showed a significant association with vitamin D status. The rs731236 GG genotype and the rs7116978 CC genotype were associated with a “vitamin D sufficiency” state. Rs731236 GG and rs7116978 CC genotypes showed a higher response to vitamin D supplementation. Transcription factor binding site prediction analysis showed altered binding sites for transcription factors according to the different rs7116978 alleles. Interestingly, the 37 SNPs previously established to play a role in vitamin D-related pathways explained very little of the response to vitamin D supplementation in our cohort, suggesting the existence of alternative loci whose number and effect size need to be investigated in future studies. Conclusion. In this paper, we present novel data on vitamin D-related SNPs and response to vitamin D supplementation demonstrating the feasibility of applying functional genomic approaches in interventional studies to assess individual-level responses to vitamin D supplementation.

## 1. Introduction

Vitamin D plays an important role in the endocrine system, and it takes part in several biological processes such as blood pressure regulation, calcium and phosphate homeostasis, nerve conduction, skeletal development, erythropoiesis, and so forth. [1,2,3]. The active form of vitamin D, 1,25-dihydroxyvitamin D [1,25(OH)2D], regulates the expression of vitamin D-related genes involved in calcium transport and bone matrix protein [4,5]. Nevertheless, vitamin D deficiency has been well documented worldwide [6]. Several factors have been shown to contribute to vitamin D deficiency, including low skin exposure to sunlight, low dietary intake of vitamin D, high body mass index (BMI), genetic predisposition, the gut microbiome, and the immune system [7,8,9,10,11]. The Middle East regions are also affected by vitamin D deficiency [3,12,13,14]. In fact, despite having ample sunshine, these regions register the highest rate of vitamin D deficiency [13]. This is partially explained by the limited sun exposure due to cultural practices. Other common risk factors in these regions include female gender and clothing style, multiparity, sedentary lifestyle, and low intake of vitamin D and calcium from the diet [14,15].

There are two forms of vitamin D: vitamin D2 and vitamin D3. Vitamin D2 mainly comes from fortified foods like breakfast cereals, milk, and other dairy items, while vitamin D3 is made by the body on exposure to sunlight [16]. In the bloodstream, vitamin D2 and vitamin D3 are converted to the major circulating form of vitamin D, which is 25-hydroxyvitamin D (25(OH)D) [17]. Serum 25(OH)D level is thus considered the best indicator of vitamin D supply to the body from both cutaneous synthesis and nutritional intake. Ethnic differences in the prevalence of common genetic polymorphisms provide an additional explanation for low vitamin D levels. Studies related to the role of the genetic background on the responsiveness to vitamin D supplementation are yet in their infancy [18,19].

Noteworthy, while current studies provide data about vitamin D deficiency, they have been mainly focused on the Western populations [20,21,22], thus their conclusions do not necessarily apply to populations with a different genetic background. Additional research is warranted to understand the role of the genetic background in responsiveness to vitamin D supplementation, especially in regions disproportionally affected by vitamin D deficiency such as the Arab populations.

To date, observational and functional studies have been performed on vitamin D in Middle Eastern countries, however, interventional studies are currently lacking. The evidence from observational studies in humans is often susceptible to bias and confounders, thus provides limited evidence for causality. We designed this interventional study to test the hypothesis whether genetic polymorphisms in genes involved in the effect and/or metabolism of vitamin D3 influence the outcome of vitamin D3 supplementation by recruiting participants with low vitamin D levels (deficient or insufficient). We believe this question to be particularly relevant since little is known about the role of the genetic background in vitamin D deficiency and/or responsiveness to supplementation in the Arab population.

When selecting SNPs (single-nucleotide polymorphisms), we aimed at choosing the ones previously reported to be associated with vitamin D-related traits in addition to a few SNPs we wanted to explore [5]. These SNPs were selected from genes associated with vitamin D and included cytochrome P450 family 2, R (CYP2R1) [5,22,23,24,25,26,27], cytochrome P450 family 24 subfamily A member 1 (CYP24A1) [22,27,28,29,30,31], the 1-alpha-hydroxylase (CYP27B1) [32,33,34,35], the 7-dehydrocholesterol reductase/NAD synthetase 1 (DHCR7/NADSYN1) [26,36], the vitamin D receptor (VDR) [37,38], and the vitamin D-binding protein GC (group-specific component) [20,21,22,26,28,39,40].

## 2. Materials and Methods

### 2.1. Study Subjects

The study was approved by Qatar University (QU) Institutional Review Board (IRB) (QU-IRB; 531-A/15) and by Sidra Medicine IRB (1705010938). A total of 100 female students from QU were enrolled in the study. All subjects enrolled were self-reported as healthy and did not have any underlying diseases. Subjects were excluded if they were taking vitamin D or antibiotics, or were suffering from any chronic disease. Subjects were excluded from the final analysis if they failed to provide the blood sample at any stage of the study (pre- or post-supplementation).

An amount of 14 mL of peripheral blood was collected from each participating subject in phase 1 before vitamin D3 supplementation, and 6 mL of peripheral blood in phase 2 after vitamin D3 supplementation. In both phases, 4 mL of blood was used for biochemical assessment. The remaining 10 mL was used for molecular analyses.

To classify the vitamin D levels S/D/I (sufficient, deficient, insufficient), pre- and post-supplementation, we followed the Endocrine Society criteria, where vitamin D deficiency is defined as serum 25(OH)D levels below 20 ng/mL (50 nmol/liter), vitamin D insufficiency is defined as 25(OH)D levels between 21 and 29 ng/mL (52.5–72.5 nmol/liter), and sufficiency is defined as serum 25(OH)D levels ≥30 ng/mL [41]. At the end of the intervention, participants were classified as either responder (R) to vitamin D supplementation (those who achieved serum levels of 25(OH) D above 20 ng/mL) or nonresponder (NR) (those whose serum levels of 25(OH) D remained <20 ng/mL) [19,42]. The biochemical measurements were performed at the Biomedical Labs in QU, while the molecular profiling was performed at Sidra Medicine.

### 2.2. Anthropometric Measures

The height was measured to the nearest 0.1 cm without shoes, using a Seca GmbH & Co. kg stadiometer. WC (waist circumference, cm) was measured midway between the lowest rib and the superior border of the iliac crest, just above the navel on standing subjects using an inelastic tape. Weight to the nearest 0.1 kg was measured in light clothing without shoes using a Seca GmbH & Co. kg weight scale. BMI (Body Mass Index) was calculated as weight (kg) divided by the squared height (m^2^).

### 2.3. Genetic Analysis of Polymorphisms Related to Vitamin D

Blood samples were collected in tubes containing ethylenediaminetetraacetic acid EDTA as anticoagulant. Genomic DNA was isolated from peripheral blood using QIASymphony DSP DNA Midi kit (Qiagen, Hilden, Germany) following the manufacturer’s recommendations. DNA quality and quantity were checked on Nanodrop 1000 (ThermoScientific, Waltham, MA, USA). The extracted DNA was used to amplify sequences containing polymorphisms related to vitamin D. These SNPs included: rs10783219, rs7139166, rs731236, rs757343, and rs4516035 in VDR gene; rs10741657, rs10766197, rs12794714, rs1562902, rs10500804, rs1993116, rs7116978, rs10877012, rs4646536, and rs703842 in CYP2R1 gene; rs11234027, rs12785878, and rs3829251 in DNCR7/NADSYN1 gene; rs1155563, rs12512631, rs16846876, rs17467825, rs222020, rs2282679, rs2298849, rs2298850, rs2882679, rs3755967, rs4588, and rs7041 in GC gene; rs17219315, rs2244719, rs229624, rs2296241, rs2426496, rs4809960, and rs6013897 in CYP24A1 gene.

SNP-specific assays were designed and ordered through the Fluidigm D3 Assay Design tool (https://d3.fluidigm.com/account/login). SNP-related sequences are reported in Supplementary Table S1. The genotypes of the polymorphisms listed above were determined by polymerase chain reaction (PCR). In the first step, two preamplification primers (locus-specific primer (LSP) and specific target amplification (STA) primer) were used to amplify the target region containing the SNP. The DNA sequences of the SNPs of interest were preamplified simultaneously in one multiplex PCR, for each sample separately, on a Veriti Thermal Cycler (Applied Biosystems, Foster City, CA, USA), with the following conditions: hold at 95 °C for 15 min, 14 cycles at 95 °C for 15 s, and 60 °C for 4 min. A second amplification was performed on the Fluidigm 96.96 Dynamic Array.

Assay mixture was prepared by mixing 3 µL of each allele-specific primer (ASP), 8 µL of each locus-specific primer (LSP), and 29 µL DNA hydration buffer. One µl of each assay mix was combined with 2.5 µL of 2 × Assay Loading Reagent and 1.5 µL of nuclease-free water. Sample mixture was prepared by combining 2.5 µL of 1:50 diluted samples from the STA reaction and 3 µL Biotium Fast Probe Master Mix. The BioMark HD dynamic array was first primed with control line fluid, and then loaded with the samples and assay mixtures via the appropriate inlets using an IFC (integrated fluidic circuit) controller. The array chip was placed in the BioMark HD Instrument. PCR was carried out using the following cycling conditions: 50 °C for 2 min, 70 °C for 30 min, 25 °C for 10 min, and 95 °C for 5 min, followed by four touchdown cycles (95 °C for 15 s, from 64 °C to 61 °C for 45 s, 72 °C for 15 s) and 34 additional cycles (95 °C for 15 s, 60 °C for 45 s, 72 °C for 15 s). Each PCR reaction used distilled water instead of DNA as negative control. Results were plotted on a two-dimensional scatter plot of the major versus the minor allele using the BioMark SNP Genotyping Analysis software version 2.1.1. Genotyping calls were assessed based on the allele discrimination plots and manually reviewed by looking at the single amplification plots. Genotyping calls were exported as a CSV file and processed for secondary analysis.

### 2.4. Data Analysis

Chi-square was used to test association and Hardy–Weinberg equilibrium (HWE) for each SNP. SNPs that did not pass the HWE criteria have been discarded from further analysis. All statistical assessments were two-sided and considered to be significant when *p*-value was <0.05. Pairwise comparisons and Bonferroni corrections were performed where relevant.

Genotyping data for the 37 SNPs under study were downloaded from the 1000 Genomes Project (http://www.internationalgenome.org) for a total of 5008 individuals (Current Build 152, released 2 October 2018, GRCh38.p12), including: Africans (N = 1322), East Asians (N = 1008), Europeans (N = 1006), South Asians (N = 978), and Americans (N = 694). The Haploview 4.2 software package was used to estimate pairwise linkage disequilibrium (LD), and to detect departure from HWE, based on the expectation maximization algorithm [43]. Among all pairs of biallelic loci, we examined widely used measures of linkage disequilibrium (LD), Lewontin’s D’ ID’I and r2 [44]. All statistical tests were based on two-tailed probability. TFBS (transcription factor binding site) prediction analysis was also performed using PROMO software, version 8.3, ALGGEN.

SNP genotypes were categorized as either major, intermediate (Inter), or minor based on the frequency. The genotype category for each SNP of each donor was used to build a color grid (pheatmap, R: A Language and Environment for Statistical Computing) [45].

## 3. Results

All characteristics of the participants are shown in Table 1. The mean age was 21 years. The mean BMI, height, waist circumference (WC), and weight were 24 kg/m^2^, 159 cm, 33 inches, and 62 kg, respectively. The participants had a mean level of ALT, AST, BUN, calcium, and creatinine in Phase 1 of 9.8 U/L, 15.1 U/L, 9.8 mg/dl, 9.2 mg/dl, and 0.46 mg/dL, respectively. The participants had a mean level of ALT, AST, BUN, calcium, and creatinine in Phase 2 of 12.9 U/L, 16.3 U/L, 12.7 mg/dL, 11.3 mg/dl, and 0.67 mg/dL, respectively. The mean level of the active form of vitamin D was 11.01 in Phase 1 and 34.3 in Phase 2. Table 1 also lists the 95% confidence interval (CI) of the mean and the *p*-value associated with the hypothesis of null distribution.

The allele frequencies of the 37 SNPs assessed in this study are reported in Table 2. Four deviations from HWE were detected (SNP IDs: rs4516035, rs16846876, rs2298849, and rs2298850). The SNPs that did not meet HWE were removed from further analyses. One SNP (rs11234027) showed only one allele in the current cohort and was also removed from further analyses. We compared the allele distribution of the 37 SNPs with that of the populations included in the 1000 Genomes Project (1000G). Several SNPs showed differences in frequencies of the Ref (reference) allele in our cohort as compared to the data from the 1000G project. However, we cannot exclude that those differences are due to a small cohort size, or perhaps due to gender specificity.

Haplotype analysis was performed by chromosome (Chr.4 for GC gene, Chr. 11 for CYP2R1 and DHCR7 genes, Chr. 20 for CYP24A1 gene, Chr. 12 for CYP27B1 and VDR genes); no significant linkage disequilibrium was detected, probably due to the small cohort size (Supplementary Figure S1).

**Figure 1:**
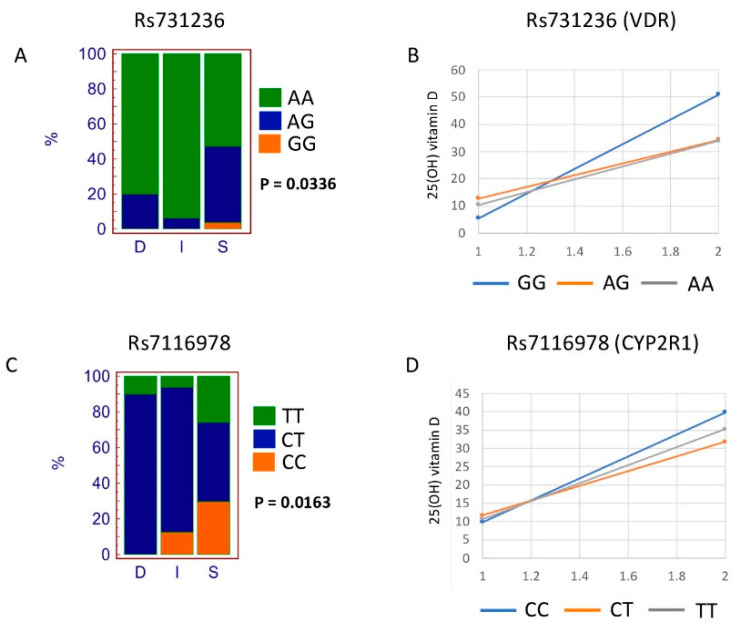
SNPs significantly associated with Deficient (D)/Insufficient (I)/Sufficient (S) classification. Genotype distribution according to Deficient (D)/Insufficient (I)/Sufficient (S) classification for rs731236 (**A**). Increment of vitamin D level from Phase 1 to Phase 2 according to rs731236 genotypes (**B**). Genotype distribution according to Deficient (D)/Insufficient (I)/Sufficient (S) classification for rs7116978 (**C**). Increment of vitamin D level from Phase 1 to Phase 2 according to rs7116978 genotypes (**D**).

We also assessed the association of each SNP with the response to vitamin D supplementation (i.e., R/NR; responder, nonresponder) and pretreatment vitamin D status (i.e., S/D/I; sufficient, deficient, insufficient) classification. Out of all the SNPs assessed, rs731236 (VDR gene) and rs7116978 (CYP2R1 gene) showed a significant association with S/D/I classification (two-tailed chi-square test, *p* = 0.0336 and *p* = 0.0163, respectively, shown in bold in Table 3). Figure 1 shows the percentage of the genotypes of the two SNPs mentioned above and according to the S/D/I classification. The rs731236 GG genotype and the rs7116978 CC genotype were associated with a “vitamin D sufficiency” state.

To better understand the association of rs731236 and rs7116978 SNPs with the vitamin D sufficiency state, we performed pairwise comparisons by creating 3 × 2 contingency tables. For rs731236, only the contingency table of AA, AG, and GG genotypes versus I (Insufficient) and S (Sufficient) classification produced a significant *p*-value (chi-square = 8.868; *p*-value = 0.0119). For rs7116978, the two contingency tables of CC, CT, and TT genotypes versus D (Deficient) and S (Sufficient) classification and of CC, CT, and TT genotypes versus I (Insufficient) and S (Sufficient) classification produced a significant *p*-value (CC,CT,TT vs. D,S: chi-square = 7.272, *p*-value = 0.0264, CC,CT,TT vs. I,S: chi-square = 6.802, *p*-value = 0.0333). Nevertheless, after applying Bonferroni correction, only the pairwise comparison rs731236 AA, AG, and GG genotypes versus I and S classification remained significant (*p*-value = 0.0357). We cannot exclude this to be due to the small cohort size.

The increase of serum 25(OH) vitamin D level (delta vitamin D values, difference between Phase 1 and Phase 2) is also shown according to the different genotypes. For SNP rs731236, genotype GG showed a higher increase as compared to AG and AA genotypes. As SNP rs7116978 is intronic, we have assessed whether the different alleles may alter the binding sites for transcription factors. The results of these analyses are reported in Supplementary Figure S2.

**Figure 2:**
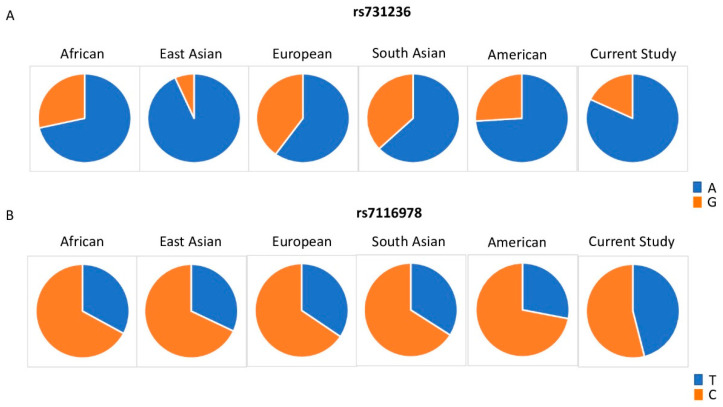
Distribution of rs731236 (**A**) and rs7116978 (**B**) allelic frequencies among world populations included in the 1000 Genomes Project and the cohort in our study.

Figure 2 shows the allelic distribution of rs731236 and rs7116978 among the populations included in 1000G and the cohort included in the current study. For rs731236, the current study displayed a lower frequency of the G allele as compared to the African, European, South Asian, and American cohorts. Allelic frequencies of rs7116978 were more similar across the populations included in 1000G. However, the current study showed a higher frequency of rs7116978 T allele as compared to all the other populations assessed. We cannot exclude the possibility that these differences are related to gender specificity; those differences may also not be represented by a small cohort as the one employed in this study.

**Figure 3:**
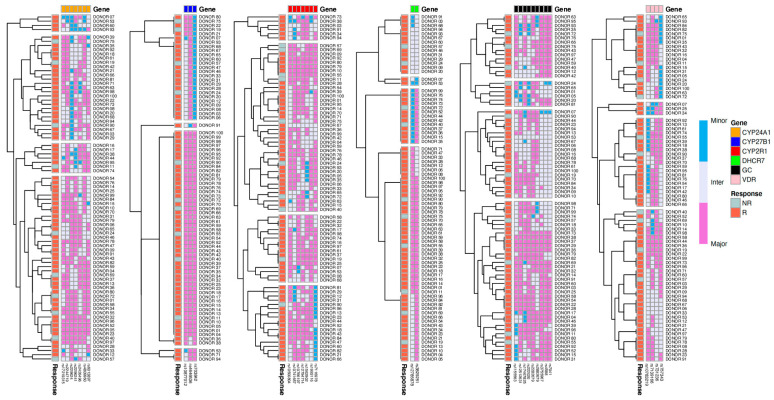
Hierarchical clustering and heatmaps based on genotyping results. Heatmaps display the hierarchical clustering (complete-linkage clustering based on Euclidean distance) of SNPs and donors based on genotype. Horizontally, at the top of the heatmaps, we added color-coded labels that correspond to the genes. The hierarchical clustering of the donors (vertical hierarchical tree, left side of heatmaps) showed that groups formed based on the genotype of certain SNPs. Genotypes are color-coded according to their frequencies, Major = pink, Inter = white, Minor = blue. SNPs that did not pass HWE were excluded from this picture. Labels of the x-axis indicate R: responder and NR: nonresponder.

We further sought to test whether the combination of different SNPs may predict R versus NR classification. Figure 3 shows heatmaps of SNPs categorized according to the genes they belong to. The heatmaps shown display the hierarchical clustering (complete-linkage clustering based on Euclidean distance) of SNPs and donors based on genotype. Horizontally, at the top of the heatmaps, we added color-coded labels that correspond to the genes. The hierarchical clustering of the donors (vertical hierarchical tree, left side of heatmaps) showed that groups formed based on the genotype of certain SNPs. No clear grouping of the participants based on their response to vitamin D supplementation was found in any of the genes assessed, suggesting that the genetic component alone does not predict responsiveness to vitamin D in our cohort.

## 4. Discussion

Vitamin D is a fat-soluble vitamin that is produced when the skin is exposed to UVB radiation [46]. Vitamin D deficiency is associated with chronic liver [47] and kidney [48] diseases.

The actions of vitamin D are mediated by several proteins. VDR (vitamin D receptor) is a ligand-activated transcription factor that acts through vitamin D response elements located near the start sites of target genes to regulate gene expression [49]. GC (group-specific component) encodes for a vitamin D binding protein that plays an important role in the transport and metabolism of vitamin D, being the major plasma carrier for vitamin D and its metabolites [50]. The vitamin D pathway also involves a series of cytochrome P450-containing sterol hydroxylases that generate and degrade the active hormone serving as a ligand for the vitamin D receptor-mediated transcriptional gene expression [51]. Among these hydroxylases, CYP2R1 is the principal enzyme carrying the hydroxylation of vitamin D to 25-hydroxyvitamin D in the liver [52]; the 1a-hydroxylation of 25(OH)D in the kidney by CYP27B1 generates the fully active vitamin D metabolite [51,52]. Cytochrome P450 family 24 subfamily A member 1 (CYP24A1) gene encodes a mitochondrial monooxygenase which catalyzes the 24-hydroxylation of 1,25-dihydroxyvitamin D3 [51].

Recent studies have suggested that SNPs within the genes above may influence the level or activity of vitamin D, but studies exploring the association of SNPs in vitamin D-related genes with the response to supplementation of vitamin D are still lacking.

The present interventional study assessed the association between SNPs previously established to play a role in vitamin D-related genes and the responsiveness to vitamin D supplementation after the intervention. Our data showed that both rs731236 (VDR gene) and rs7116978 (CYP2R1 gene) have a significant association with the vitamin D status, where the rs731236 GG and the rs7116978 CC genotypes were associated with a “vitamin D sufficiency” state. In addition, rs731236 GG and rs7116978 CC genotypes were associated with a higher response to vitamin D supplementation. With the exception of rs731236 and rs7116978, the remaining SNPs previously established to play a role in vitamin D-related pathways explained very little the response to vitamin D supplementation in our cohort, suggesting the existence of alternative loci whose number and effect size warrant future studies.

Rs7116978 is located in an intronic region. Whereas the recognition of functional variants in the coding region is relatively simple, detecting changes in the noncoding region is more challenging due to the lack of a clear connection between nucleotide differences and regulatory functions. Some introns have been shown to contain transcription factor binding sites [53,54] and to influence gene expression by several known and unknown mechanisms such as intron-mediated enhancement [55,56]. In this study, we speculated that the two alleles of rs7116978 may change potential binding sites for different transcription factors. We have performed TFBS (transcription factor binding site) prediction analysis in silico to verify this hypothesis. The two alleles were predicted to modify transcription binding sites of several transcription factors, including transcription factor 4 (TCF4) and the human glucocorticoid receptor (GR) (Supplementary Figure S2). Several studies have shown the link between these transcription factors with vitamin D metabolism. Beildeck et al. found that 1,25(OH)2D3 induces the expression of TCF4 in several human cell lines via a VDR-dependent mechanism [57]. As per the proposed model, TCF4 upregulation would enhance the repression of β-catenin/TCF target genes. With regards to the human glucocorticoid receptor (GR), cross-sectional studies have shown that the chronic use of glucocorticoids is associated with low levels of 25(OH)D [58]. It appears that the glucocorticoids can upregulate the renal expression of CYP24A1 which in turn catabolizes 25(OH)D and 1,25(OH)2D to water-soluble inactive agents, thus mediating deficiency of vitamin D [58,59]. Another link between the use of glucocorticoids and vitamin D is that 1,25(OH)2D/VDR and glucocorticoids/glucocorticoid receptor (GR) intracellular signaling pathways cross-talk so that the increased levels of vitamin D may enhance the responsiveness of certain target cells to glucocorticoids [60,61,62]. Glucocorticoids with their cognate receptors translocate from the cell cytoplasm to the nucleus where they bind to glucocorticoid response element (GRE) to regulate gene transcription. As VDR and GR share some coactivators, VDR may promote individual gene transcription induced by GR; additionally, it has been reported that vitamin D may upregulate the binding of GR to GRE [60]. These may result in vitamin D enhancing the cellular responsiveness to glucocorticoids. Functional studies are warranted to understand the importance of rs7116978 polymorphism as a potential regulator of gene expression.

The vitamin D molecular pathway is a complex process that varies across individual genetic profiles and according to their health status. As this current topic is still in its infancy, additional studies are warranted to elucidate how genetic variation contributes to vitamin D supplementation outcomes.

## 5. Conclusions

In conclusion, the present interventional study shows novel data about the association of vitamin D-related SNPs with responsiveness to vitamin D supplementation in individuals of Arab ancestry. Our findings should be validated in additional studies employing a cohort of a bigger size.

## Figures and Tables

**Table 1:** Characteristics of the participants (N = 100).

	Mean	95% CI	Normal Distribution
Age	21.173	20.803–21.542	0.0004
BMI (kg/m2)	24.344	23.298–25.390	<0.0001
Height (cm)	159.407	158.252–160.563	0.7273
Phase 1 ALT (U/L)	9.825	8.798–10.852	<0.0001
Phase 1 AST (U/L)	15.072	13.967–16.176	0.0015
Phase 1 BUN (mg/dl)	9.821	9.195–10.447	0.0109
Phase 1 Calcium (mg/dl)	9.171	8.882–9.459	0.6413
Phase 1 Creatinine (mg/dl)	0.463	0.437–0.490	0.4344
Phase 2 ALT (U/L)	12.948	11.525–14.371	<0.0001
Phase 2 AST (U/L)	16.344	15.118–17.571	0.0016
Phase 2 BUN (mg/dl)	12.664	11.883–13.446	0.1309
Phase 2 Calcium (mg/dl)	11.324	10.996–11.652	0.0367
Phase 2 Creatinine (mg/dl)	0.674	0.639–0.708	0.0009
Vitamin D Total Phase 1	11.014	9.989–12.038	0.1375
Vitamin D Total Phase 2	34.332	31.430–37.234	0.8644
Waist Circumference (inch)	33.083	31.093–35.073	<0.0001
Weight (kg)	62.149	59.047–65.251	<0.0001

**Table 2:** Allele frequencies of the SNPs assessed in this study across the 1000G populations and the cohort from the current study. HWE: Hardy–Weinberg equilibrium; NA: not available. *, SNPs that did not pass HWE. All alleles in the table below are reported in the forward orientation.

SNP ID	Gene	Chr.	Allele	African (N = 1322)	East Asian (N = 1008)	European (N = 1006)	South Asian (N = 978)	American (N = 694)	Current Study Cohort (N = 89)	HWE *p*-Value
rs10783219	VDR	12	Ref (T)	0.023	0.441	0.332	0.33	0.51	0.55	0.29
			Alt (A)	0.977	0.559	0.668	0.67	0.49	0.45	
rs7139166	VDR	12	Ref (C)	0.963	0.981	0.577	0.8	0.72	0.73	0.17
			Alt (G)	0.037	0.019	0.423	0.2	0.28	0.27	
rs731236	VDR	12	Ref (A)	0.715	0.933	0.6	0.63	0.74	0.82	0.53
			Alt (G)	0.285	0.067	0.4	0.37	0.26	0.18	
rs757343	VDR	12	Ref (C)	0.945	0.783	0.854	0.89	0.85	0.6	0.07
			Alt (T)	0.055	0.217	0.146	0.11	0.15	0.4	
rs4516035	VDR	12	Ref (T)	0.963	0.981	0.578	0.8	0.72	0.6	0.05 *
			Alt (C)	0.037	0.019	0.422	0.2	0.28	0.4	
rs10741657	CYP2R1	11	Ref (A)	0.219	0.319	0.381	0.36	0.28	0.382	0.37
			Alt (G)	0.781	0.681	0.619	0.64	0.72	0.618	
rs10766197	CYP2R1	11	Ref (G)	0.896	0.643	0.526	0.55	0.64	0.15	0.97
			Alt (A)	0.104	0.357	0.474	0.45	0.36	0.85	
rs12794714	CYP2R1	11	Ref (G)	0.897	0.632	0.553	0.56	0.49	0.17	0.67
			Alt (A)	0.103	0.368	0.447	0.44	0.51	0.83	
rs1562902	CYP2R1	11	Ref (C)	0.477	0.379	0.441	0.43	0.33	0.74	0.25
			Alt (T)	0.523	0.621	0.559	0.57	0.67	0.26	
rs10500804	CYP2R1	11	Ref (T)	0.897	0.63	0.552	0.55	0.48	0.8	0.77
			Alt (G)	0.103	0.37	0.448	0.45	0.52	0.2	
rs1993116	CYP2R1	11	Ref (A)	0.219	0.32	0.388	0.35	0.28	0.77	0.74
			Alt (G)	0.781	0.68	0.612	0.65	0.72	0.23	
rs7116978	CYP2R1	11	Ref (T)	0.329	0.32	0.344	0.34	0.28	0.46	0.37
			Alt (C)	0.671	0.68	0.656	0.66	0.72	0.54	
rs10877012	CYP27B1	12	Ref (G)	0.921	0.357	0.683	0.49	0.74	0.85	0.95
			Alt (T)	0.079	0.643	0.317	0.51	0.26	0.15	
rs4646536	CYP27B1	12	Ref (A)	0.756	0.354	0.681	0.42	0.72	0.03	0.37
			Alt (G)	0.244	0.646	0.319	0.58	0.28	0.97	
rs703842	CYP27B1	12	Ref (A)	0.7	0.354	0.68	0.42	0.71	0.15	0.1
			Alt (G)	0.3	0.646	0.32	0.58	0.29	0.85	
rs11234027	DHCR7/NADSYN1	11	Ref (G)	0.651	0.696	0.808	0.65	0.76	0	NA
			Alt (A)	0.349	0.304	0.192	0.35	0.24	1	
rs12785878	DHCR7/NADSYN1	11	Ref (G)	0.83	0.62	0.299	0.85	0.55	0.57	0.38
			Alt (T)	0.17	0.38	0.701	0.15	0.45	0.43	
rs3829251	DNCR7/NADSYN1	11	Ref (G)	0.743	0.702	0.809	0.65	0.77	0.15	0.94
			Alt (A)	0.257	0.298	0.191	0.35	0.23	0.85	
rs1155563	GC	4	Ref (T)	0.943	0.664	0.756	0.68	0.81	0.6	0.14
			Alt (C)	0.057	0.336	0.244	0.32	0.19	0.4	
rs12512631	GC	4	Ref (T)	0.647	0.775	0.653	0.74	0.57	0.75	0.19
			Alt (C)	0.353	0.225	0.347	0.26	0.43	0.25	
rs16846876	GC	4	Ref (A)	0.853	0.74	0.702	0.7	0.78	0.403	0.04 *
			Alt (T)	0.147	0.26	0.298	0.3	0.22	0.596	
rs17467825	GC	4	Ref (A)	0.946	0.739	0.751	0.7	0.79	0.72	0.32
			Alt (G)	0.054	0.261	0.249	0.3	0.21	0.28	
rs222020	GC	4	Ref (C)	0.633	0.416	0.162	0.13	0.21	0.73	0.17
			Alt (T)	0.367	0.584	0.838	0.87	0.79	0.27	
rs2282679	GC	4	Ref (T)	0.95	0.739	0.753	0.7	0.79	0.12	0.79
			Alt (G)	0.05	0.261	0.247	0.3	0.21	0.88	
rs2298849	GC	4	Ref (A)	0.573	0.599	0.801	0.84	0.78	0.6	0.05 *
			Alt (G)	0.427	0.401	0.199	0.16	0.22	0.4	
rs2298850	GC	4	Ref (G)	0.974	0.74	0.764	0.71	0.8	0.75	0.02 *
			Alt (C)	0.026	0.26	0.236	0.29	0.2	0.25	
rs2882679	GC	4	Ref (C)	NA	NA	NA	NA	NA	0.74	0.99
			Alt (T)	NA	NA	NA	NA	NA	0.26	
rs3755967	GC	4	Ref (C)	0.946	0.739	0.752	0.7	0.79	0.19	0.38
			Alt (T)	0.054	0.261	0.248	0.3	0.21	0.81	
rs4588	GC	4	Ref (G)	0.933	0.739	0.752	0.7	0.79	0.77	0.29
			Alt (T)	0.067	0.261	0.248	0.3	0.21	0.23	
rs7041	GC	4	Ref (A)	0.906	0.7	0.417	0.46	0.46	0.75	0.16
			Alt (C)	0.094	0.3	0.583	0.54	0.54	0.25	
rs17219315	CYP24A1	20	Ref (A)	0.997	1	0.982	0.98	0.99	0.22	0.43
			Alt (G)	0.003	0	0.018	0.02	0.01	0.88	
rs2244719	CYP24A1	20	Ref (C)	0.239	0.129	0.428	0.29	0.52	0.15	0.97
			Alt (T)	0.761	0.871	0.572	0.71	0.48	0.85	
rs229624	CYP24A1	20	Ref (G)	0.893	0.969	0.815	0.8	0.9	0.53	0.35
			Alt (A)	0.107	0.031	0.185	0.2	0.1	0.47	
rs2296241	CYP24A1	20	Ref (G)	0.508	0.594	0.467	0.58	0.58	0.54	0.19
			Alt (A)	0.492	0.406	0.533	0.42	0.42	0.46	
rs2426496	CYP24A1	20	Ref (T)	0.432	0.357	0.324	0.35	0.24	0.74	0.99
			Alt (G)	0.568	0.643	0.676	0.65	0.76	0.26	
rs4809960	CYP24A1	20	Ref (T)	0.905	0.78	0.769	0.75	0.79	0.82	0.84
			Alt (C)	0.095	0.22	0.231	0.25	0.21	0.18	
rs6013897	CYP24A1	20	Ref (T)	0.74	0.851	0.758	0.67	0.63	0.72	0.37
			Alt (A)	0.26	0.149	0.242	0.33	0.37	0.28	

**Table 3:** Association of genotypes with R/NR and S/D/I (chi-square). Significant *p*-values are in bold.

SNP ID	Gene	*p*-Value (R/NR)	*p*-Value (S/D/I)
rs10783219	VDR	0.6071	0.5330
rs7139166	VDR	0.4249	0.6209
rs731236	VDR	0.5133	**0.0336**
rs757343	VDR	0.6023	0.6926
rs10741657	CYP2R1	0.3748	0.4870
rs10766197	CYP2R1	0.7235	0.3820
rs12794714	CYP2R1	0.7221	0.8857
rs1562902	CYP2R1	0.3136	0.1389
rs10500804	CYP2R1	0.7443	0.8867
rs1993116	CYP2R1	0.6900	0.6161
rs7116978	CYP2R1	0.0727	**0.0163**
rs10877012	CYP27B1	0.7108	0.3583
rs4646536	CYP27B1	0.8004	0.8265
rs703842	CYP27B1	0.7845	0.7230
rs12785878	DHCR7/NADSYN1	0.9386	0.9820
rs3829251	DHCR7/NADSYN1	0.7488	0.4279
rs1155563	GC	0.9104	0.9904
rs12512631	GC	0.3288	0.1126
rs17467825	GC	0.0661	0.1188
rs222020	GC	0.4039	0.4397
rs2282679	GC	0.9068	0.7540
rs2882679	GC	0.4395	0.6346
rs3755967	GC	0.8826	0.5654
rs4588	GC	0.2287	0.1922
rs7041	GC	0.1961	0.2328
rs17219315	CYP24A1	0.6713	0.3635
rs2244719	CYP24A1	0.7235	0.3820
rs229624	CYP24A1	0.8586	0.8796
rs2296241	CYP24A1	0.3863	0.4631
rs2426496	CYP24A1	0.4395	0.6346
rs4809960	CYP24A1	0.5668	0.1768
rs6013897	CYP24A1	0.7249	0.9156

## Supplementary Materials

The following are available online at https://www.mdpi.com/2072-6643/12/9/2608/s1, Figure S1: D’ pairwise linkage disequilibrium (LD) plots of analyzed polymorphisms in Chromosomes 4, 11, 20 and 12, Figure S2: Transcription Factor Binding Sites Prediction for intronic SNP rs7116978. Table S1: SNP IDs and related sequences.

## Abbreviations

**ALT**	Alanine Aminotransferase
**AST**	Aspartate Aminotransferase
**BMI**	Body Mass Index
**BUN**	Blood Urea Nitrogen
**HWE**	Hardy–Weinberg Equilibrium
**IFC**	Integrated Fluidic Circuit
**IRB**	Institutional Review Board
**LD**	Linkage Disequilibrium
**LSP**	Locus-Specific Primer
**PCR**	Polymerase Chain Reaction
**SNP**	Single-Nucleotide Polymorphism
**STA**	Specific Target Amplification
**TFBS**	Transcription Factor Binding Site
**PCA**	Principal Component Analysis
**QU**	Qatar University
**WC**	Waist Circumference
**1000G**	1000 Genomes
