# Supplementary material for: The Role of Polymorphisms in Vitamin D-Related Genes in Response to Vitamin D Supplementation
Source: Nutrients. 2020 Aug 27;12(9):2608. doi: 10.3390/nu12092608 (PMC7551134; doi:10.3390/nu12092608)
Supplement: Supplementary file 1 [file nutrients-12-02608-s001.pdf]

Supplementary Table 1. SNPs ID and related sequences.

Target Name	Sequence
rs2298849	ATTCTTTAATGTTCACAAGGGACATCTGCATTTATCCTGCTTTGTAAAATTAAGGTTTTCATTTAGTGCTGTCAGTTAACAGCCTCACCTAATTCGTACA[C/T]TATTATTCATAATAGTAATGTGTTTTGCCAGTGGAATAAAATGTGTGTTCCCAAGAATTATCAGTTATTAACTGTAACATGAATAACCTTACTCTTGACT
rs12512631	TACTACTATTGTTAATTGCAAACTAGAATTTTCCAGAATGCTTTAGCTATGAAACTAGTAGCCTTGTGGTGGTGGTTGTCATGGAAGAAACAATTAGATG[C/T]TTTCTTGGCCTAATAGAGAGAAAAGAATATTTCAGGTCCTGGAAAAAATGGACAAGGATAAAATAGCCTCTGGGAAAGAGAATCTTTTGACCTGATTATC
rs16846876	TTTTCGTTTGTGGATTTTGTTGTATTTTCGCAACTTTAATGCCTTGTCAGTTTTAGAATTGTAAATATATTATCCCTACCTGCACATGTCTGTGAACTTT[A/T]TTCATGATGTCTTTTGTTGAACAGAACTCCTAAACTTGATAATAAAATTMATCAATATTTTGCCTTATGATTTGTCCTCTTTGAATTTTTCTTAGGTAGG
rs17467825	AAATGGAACAAATGACATCATTATGCATATTAGATCTAACATTAAAACTCTTTTACTAAAATTACAATATTTCTGTCAGCGATTCTTAATATAAGAAAAA[A/G]TGGTGAAATGTGTTTAGAGTGTGCTGGAAAATATATCCATGGGAACTATGAAAACCCTACTTCAGTGAATTGTATATTTATTGACTACTTTGTGACAAWA
rs3755967	TGATGAAGGCCTACCAATGTTCAATTATTTCAGGAATAGTTACATGGCTTTGAAAAAGCAAATTATACCTTGTTCACAAAGTTATAGGTCTGAGGTACTT[A/G]ATATCCAGAAATAATGAGACCACAACTTTACCACTTATCTTAGGTTTCCAGAACACAACCAACTCATTGCAGTTCCTGGGTTCCAGGGAGAATTTTGTAG
rs2298850	AAAAGACCCATTTGCCTACAGAAAAACCTATAGTTACAAAATTAGACTTGAAATCTTCTTTTCTCTTTCTACCATCATTCAGGCAGGGCATCCCTTTGGG[C/G]TCTTCTGAGAACTCATTACTATGCTTCCTTTAAAATGTTTCAACAGACTGCTAGTGAGCATCAGATATGATTTCCCAAAGTATTCCAAAAGTCATTGTAA
rs731236	GGTGGGATTGAGCAGTGAGGKGCCCAGCTGAGAGCTCCTGTGCCTTCTTCTCTATCCCCGTGCCCACAGATCGTCCTGGGRTGCAGGACGCCGCGCTGAT[C/T]GWGGCCATCCAGGACCGCCTGTCCAACACACTGCAGACGTACATCCGSTGCCGCCACCCGCCCCCGGGCAGCCACCTGCTCTATGCCAAGATGATCCAGA
rs10783219	TGCCTACCCTTGCCTTCCTGGCTGCTTACCTGCTTTACAGCCAGACTTTCTGTGGGATAGTGTGGTCCCCAGAGGTCTGGGAAGCTGGATGAGCAAAATG[A/T]GGAGGCTGTAGATATGGAGGAAGAGGGAAGAGGAAACGTGAGGCCCAGTCTTGGCTCCACTATGAACCCCAATTTCCCCAGGGGAAGCTGAAGAGGAGTT
rs7139166	AGAAACAGATGAAGTGCCCCTTTAGAGGTCCTGCCAGTCTGATGGATGGATTCTCTACCTGTAATGAAAGTAATAGGAAGGATCCCCTTGCCCAAAGCAT[C/G]GTGGGAAAGCTAGCGGTGATTCTTGTGGGAAGAAAAGCATAAGAGGAAATTTCCAACCACCAATACCTTGGGACATTCTCTGCAAGTCCCATGAGCCTCT
rs4516035	GGAGAATGTATAAATAAATAGTAATTACAACATAATCTTCTGGAATAGAAATGCTCACAAAATCATCCAGCAGTCGATGACCTCCTTTAGCCAGGGAAGA[C/T]ATTGCTATTCGCCTCTTACAGAGGAGGAAACCATGGCTCAGAGGGACAAGGTGAAAGGGTGGGAAATTGAGGCATAGAAAGTTTAAGTGATATGCCCAAC
rs757343	AGGTTTCTTGCGGGCAGGGTACAAAACTTTGGAGCCTGAGAGATGGTTCTGCCTATATAGTTTACCTGATTGATTTTGGAGGCAATGTGCAGTGACCCTT[A/G]ACCTCTTCCGCTGGTTAGAGGTGAGAAGAGGGAGAAAAGGCCGAAGAGGAAGTTATTGTGACCTTGGGGACATGATGTCGGTGATGAGGTCCAAAGAGGG
rs703842	AAGAATACAAGATCCCGTCCTCCAGGCAGTGACCTCCCAAACCAGCCTGCCTGGTCACTGACTGCTTACTCTACCTTAGCTGGACCTCGTCTCCCAGGGA[C/T]TAGAGAAAAGAGCAGGAGTCCTGGGTCTTCCCAGTTGAGACTGCTGGAGCTGAGACACAGTACTCTCTTAAAGAAGGTGGGGAGCTGCCCAGGGCAGAAC
rs4646536	CAGGGAGATAGCAGAGAAAAATGGCCCTCTACTCCTGGCCAAAAAGGGTTTGGAAGTTGGAAACAATGAGAAGGGGGCTGCAGCCCCTAGCCTCATCTTG[C/T]TGTCTCCATTTTGTGCTTTGCAACCTAGACTGTACYCTGTGGTACCTGGAAATTCTSRTGTCCCAGACAAAGACATTCATGTGGGTGACTATATTATCCC
rs10877012	AGCCCGGGAGGTCAAGGCTGCAGTGAGTTATGATTGTACCACTGCACTCCAGCCTAGGCAACAGAGAGAGGGCCTGTCTCTAAAAAAAATTAAAATAAAA[G/T]AATCTCCCACAGTTTACCTCTCTGCTCCTTACTCCCTCATTCCCCACTGCAACTCTATCAGGCTCTGTGCCAGTCAGCTAGCTCTGAAGTTCTGGAATTG
rs1562902	CTACAAGTGTGCATAACATAGGAAATGTGATCAGTCTTGGAGGTCAGTAATTGTAGTCTGCACCAAACAACCAGCTTATATCCAGGGACTCCCCCATGAT[C/T]GTGTTCATGGAAGATGTTACTACAATTCTTCCCTATCAACTGGTCTCATATGGACACTGAGTGAAGGGAAAGGAAAGTAACAAAATCCTCTRTTTAAAAT
rs10500804	CATGGCACTTATTTAACACTATCTTGAACCAGAAACATTCATATTCTTGTCTTCTGCCTGCATGCTAAATTACAAACTCCTTAGGAGCAGTGATTTCATC[G/T]TTTTCATCTTTGAATCCTTACACAGAATCTCATTCAGTGCTGTGCATAAAGTTCTCAATAAATGCTGTAAAAATAAATTAATACGCTGTCAATTCTACTT

rs10741657	AGAGCAATGACATGGAAATACAATAATTTTCAATAATCAGAAGCAAACAAAAAGTGCGTTATAGAAAACGCCTGGTGG TTGGGGAGATACTTTAGCAGGC[A/G]AGGGCTGTCAAGGAAAGTCTTATTAGGAAGATGACATTGGAGCTGAGATCAA CTGCTAAAGAAGTGTATTTGTTTCCTACTACTGTTGTAATAAATTACC
rs10766197	AATAGCAGTCCCTTTAATAGAAGGCTTTTGGCTGTCTCCTTCAAAAACCATAATCTCTGTACAATTTGGAACACTCCAGTT AATTAGAGATCTTTAAACT[A/G]TGCCAAGATACAGAAAGGACCAAGCTTTAAAAAAATATTGTCTTCCACATGGATTGG TCTAAAAGCAGTATTTTAACCTAAGCTYTGAAATACAGTTTTAC
rs12794714	AGCCCGGGCCAGCCTGGCGGCCCTCCCTGCCCGGGGCCCGTCGGGGCTGTACCTCTCCGTACACCTGGCTCTGCTTTCT CATGTAGACATGGGGAAGCTC[A/G]GATGAGGCTGCCAGGGAATAGATGTTGCCGATAAATGGCAGCCCCGGCGGCC CCGGGGGGAAGCCCATCGGCCGCCTCTGCTTCAGCAGCTGGCGGACCC
rs7116978	TTAAAATTATTTCATAGTTGTATAGTCTTATTCACTTTAATTTTTAAATAAAATTCATTTAAGTGCTTAAGTCACCTTTTAT AGGTAAAAGATTATCTAA[C/T]ACAATACTTTGAATGTGTATTCCTTAAAGACTTCTATTTTGAAGTCTCTCAAAAAAATG CCATATGAATGGGGCTCTAGACTTCCTATTAAGTTTCAATC
rs1993116	TTATTGAGAACTTTATGCACAGCACTGAATGAGATTCTGTGTAAGGATTCAAAGATGAAAAMGATGAAATCACTGCTCC TAAGGAGTTTGTAATTTAGCA[C/T]GCAGGCAGAAGACAAGAATATGAATGTTTCTGGTTCAAGATAGTGTTAAATAAG TGCCATGGGAGCTTCTAAGCTCCCATGTGTGGTTCAAAGCAGAGAG
rs3829251	TGTGCTCACCAGGGTGTGCAGCAGGTCCTGGGAGTTGGCTGCAGGCTGGCTCCTACCCCTCCTCCTTTCTCTTCCCCTGC CCTCACTGCCTCTTCCACGG[A/G]CAGTTCTCTATGTGAGGACAGTGGAAGGAGGGCACCTTTCCRGAGAAGRAAGACT GGGTTCTATCTAGAATATTCCATCCATAATGGCTCCTAGGAGAAA
rs12785878	TGTTCTTTAATTTGTCTTCATTTGATTGATTCTTTAAGAAATTCTTCACCTAAGTGCCAAGGGATCTAGGGTTCTGGGCTG TCTGATATCACAAAGCTTC[G/T]ATCCTCTCCTGGCCCCGTGGCCGGATCTTCTCCTGGGCTGGACTCAAGGCCTGATCG TCTCGTGAGCCTGACCTCTCCTGGGCTGGTCCGGGGGCCTGAT
rs11234027	CTGGTGACATGTTGCTGTATTCTTGGGTGAGAATGCTRAGGACATGCTGCTGTATTCTTGGGCGAGTACATTGGTGACA TGCTGCTGTGTTCTTAGGTGA[A/G]TACACTGGTGAAACAATGCCCAATCKCACGGCCAAGAGWTTTGAGGAGCAGGG ACATCTGGTAAGCAGACTTCAGTTTCTTTTCAGTAATGTGCCCCTCT
rs2244719	AAGCCATTCACCAGAGGAAGAGAAGGGAAGGGAATAATTATGTTCTAAACACCCATTACACGCCAGGTACTGGGCTAG CACTTCATATAGGGTTTTTTTT[C/T]CTTCCATTTCATCATTTTATTTAGAAGGAACCTTTTTACAAATAAAATTCTCAACTC TTCATTTCAAAACTTTTTACCTAGAGAAAAGTTGAAGGAATGG
rs2296241	ACGACAACTTACTTTCAAACGACCATTTGTTCAGTTCGCTGTACAAGTCTTCAACGTGGCCTCTTTCATCACAGAGCTCAT CTATTCTGCCCATAAAATC[A/G]GCCAAGACCTTCAAAGAAAACAACCGCAAAAGACACATTTTAAAGCCGTTGAAGGA AAACAAAACTTTAAAGCTCACTGAGCTAACTTCAGGTCTTGCTA
rs2426496	GCTTGGGCTGAAACCATGGCTCCACCACTCATTAGCTGTGTGATCTTGGGCAAGTTATTTAACTTCTCTGAGTCTAGTTT CCTTAATGGTAAAATTGGAG[G/T]TTACTATACCTGTGTCTCAGGAAGGTCAAGATAAATACTAAAAATATTAGACAAA ACACGATTGTCTTCACTAAAATCTGTTTGGCCTCTGTACTTGCTT
rs4809960	GGCATAACTTAAGCACCTAAAAGAAGCATTAAAAGGGCTGCTCTGGCCTTTCCCTAGGCAGCAATAATGCCTGTTTACA AAAGAGTTGTCAAAAGAGCCA[C/T]GTTTTATCCGCAAAATCACCTGCAAAATCAGTGAGCAAGTCTGTGACGACAACT TACTTTCAAACGACCATTTGTTCAGTTCGCTGTACAAGTCTTCAAC
rs4588	AAATTTGTATAAAATAAATACATGTAGTAAGACCTTACATTTAAATGGTTTTTCAGACTGGCAGAGCGACTAAAAGCAA AATTGCCTGAKGCCACACCCA[A/C]GGAACTGGCAAAGCTGGTTAACAAGCACTCAGACTTTGCCTCCAACTGCTGTTCC ATAAACTCACCTCCTCTTTACTGTGATTCAGAGGTAGGAAAATGT
rs17219315	TTAGCATCAGAATATTTGGGATAAAATAGTGATCATCTTTTGACAATAGTTTGACAATAGGATGAAAAAGCACCTTTCCT CCTAGTCAAAGATTGCACCA[A/G]AAAGTTGAAGTCCAGATAATTTGGGTTCAGGGATTTTGAGGTGTCCTTGCAGGGA GTGGGGCTGGGAGGCCTGGGGACAATTCCTAGCGGTAAAAGGGGG
rs6013897	CTCTGCCACTTACCTACTGTGTATCTTTGGGTAGGTTACTTCTGCCTCTGTCTTGGTTTCCTCATCTGTAAAAGGGGGATA ATGAAAGTACCTACTTCAG[A/T]GGATGATTACACTTCCTTCACATGCATTTACGAGTTTTCTGAACATATAATCCTGTTG AGATCCATGTGGACTTGATGATTCTAATTATTTGTCATGCAT
rs7041	TTTTGCATTAGAAATTTGTATAAAATAAATACATGTAGTAAGACCTTACATTTAAATGGTTTTTCAGACTGGCAGAGCGA CTAAAAGCAAAATTGCCTGA[G/T]GCCACACCCAMGGAACTGGCAAAGCTGGTTAACAAGCACTCAGACTTTGCCTCC AACTGCTGTTCCATAAACTCACCTCCTCTTTACTGTGATTCAGAGG
rs229624	CATGCCTCCTCTTCATGTCGTTGATGTACCAGGCCACACAGGTCATGGTGTCAATGGCATCCTCCACCACCTCAAAKCCA TCCTCTTCTTCATCAAAATG[C/T]TTGGCAATTTCCTGCGCAGAAGATAGACGCTTAGGTGCCCCTGACCCGAAGGCCCT GCCCTCCCCAGGCCCTAGGTGCTTGGGAGGACCGAGGGGCTACC

rs222020	GAATAACAAGGGAGAGAGATCACAGAAGAACACAGTAAGAGAAAACGGCAGCTTTACACATCTTGGTAACCAGAGGAGACAACCTTGGCTCACCATGCTG[C/T]CCATTGAATTTTTTGNCCATTGAGTTGAGTTTTTCCTGCTGCTATCTCTCTTACTAGCTTCTCCTTTCCTGTGTTAGCCCTGGAGGAGGTAGAAGCAGCC
rs2882679	TGATTTAGGACCATACCACGGAGAGACACAAAGTGGTACCAGAGACCAGCTCATTAAGTTTACCATATACAACTATGGTAATTTGTTTTTAAAAGATATT[C/T]TAAAACAGGTAAGTCAAAGTTTCACATATGGAAATATGTCCAAATTAAAATGTGCTTGTCTTCCTGAAGAGTCTGGGTCATTCATTAAAAAAAAAAAAAA
rs1155563	GAACTGATAATATCAAAATAACAAATTTACTTTCGTGTAGTAAAATTTGAAGACTGAAATGGTTATTCTAAGACTGTGCTCTTGCTATTGTANTTTTTAA[C/T]AGATAAAAAAATCACAGTTATTTCCATATAAAAATATTTTCACTCATAAATGGGAGTCGAACAGTGAGAACACATRGACACAGGGAGGGGAACATCACAC
rs2282679	TTCTGACCTTGTGATCCACCCACCTCGGCCTCCCAAAGTGCTGGGATTACAGGCGTGAGCCACCATGCCCAGCAAATCTCTGTCTCTTAATTATCTCACA[A/C]AGCCAGGTATTTTTTATTGTTAGCTTTGGAAGCAAGTAGTAGTCCCTCTGGGTTGCCAAAAACATAAATGAATTGGCTCACAGTGTAGAATCAGATTGGG

Supplementary Fig. 1

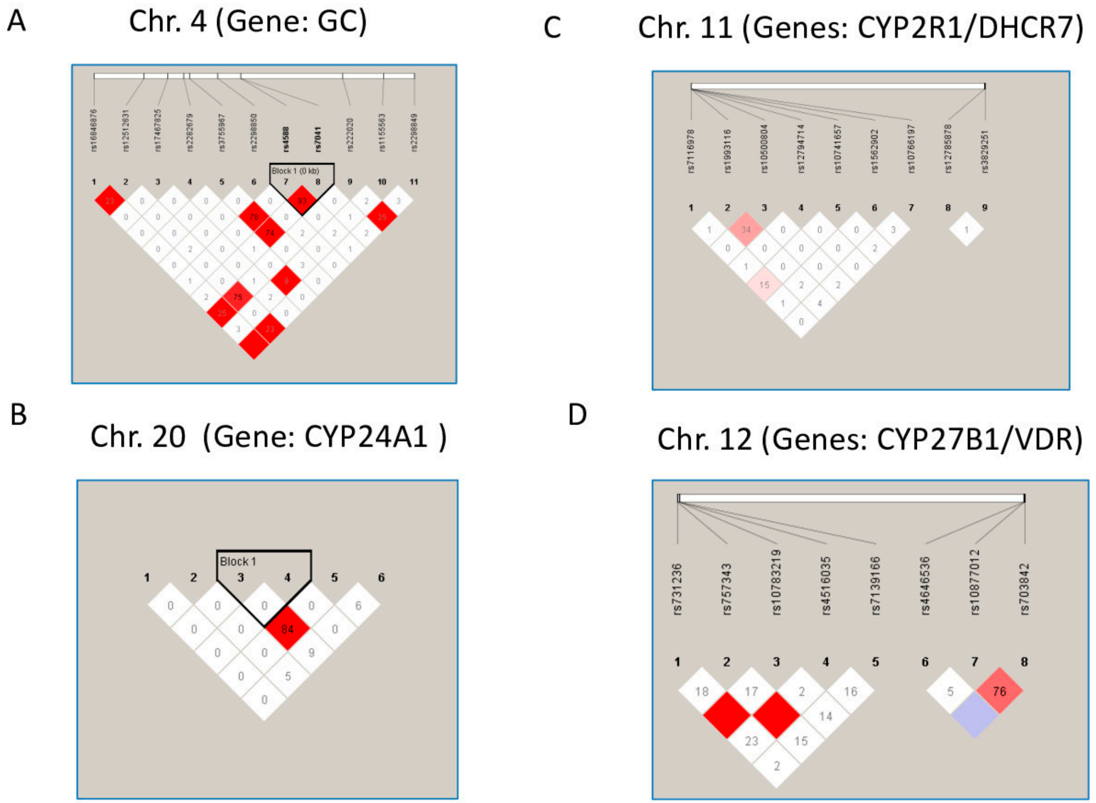

Supplementary Fig. 1. D’ pairwise linkage disequilibrium (LD) plots of analyzed polymorphisms in Chromosomes 4, 11, 20 and 12. The red shaded boxes correspond to the paired D’ values between the SNPs.

Supplementary Fig. 1. D’ pairwise linkage disequilibrium (LD) plots of analyzed polymorphisms in Chromosomes 4, 11, 20 and 12. The red shaded boxes correspond to the paired D’ values between the SNPs.

Supplementary Fig. 2

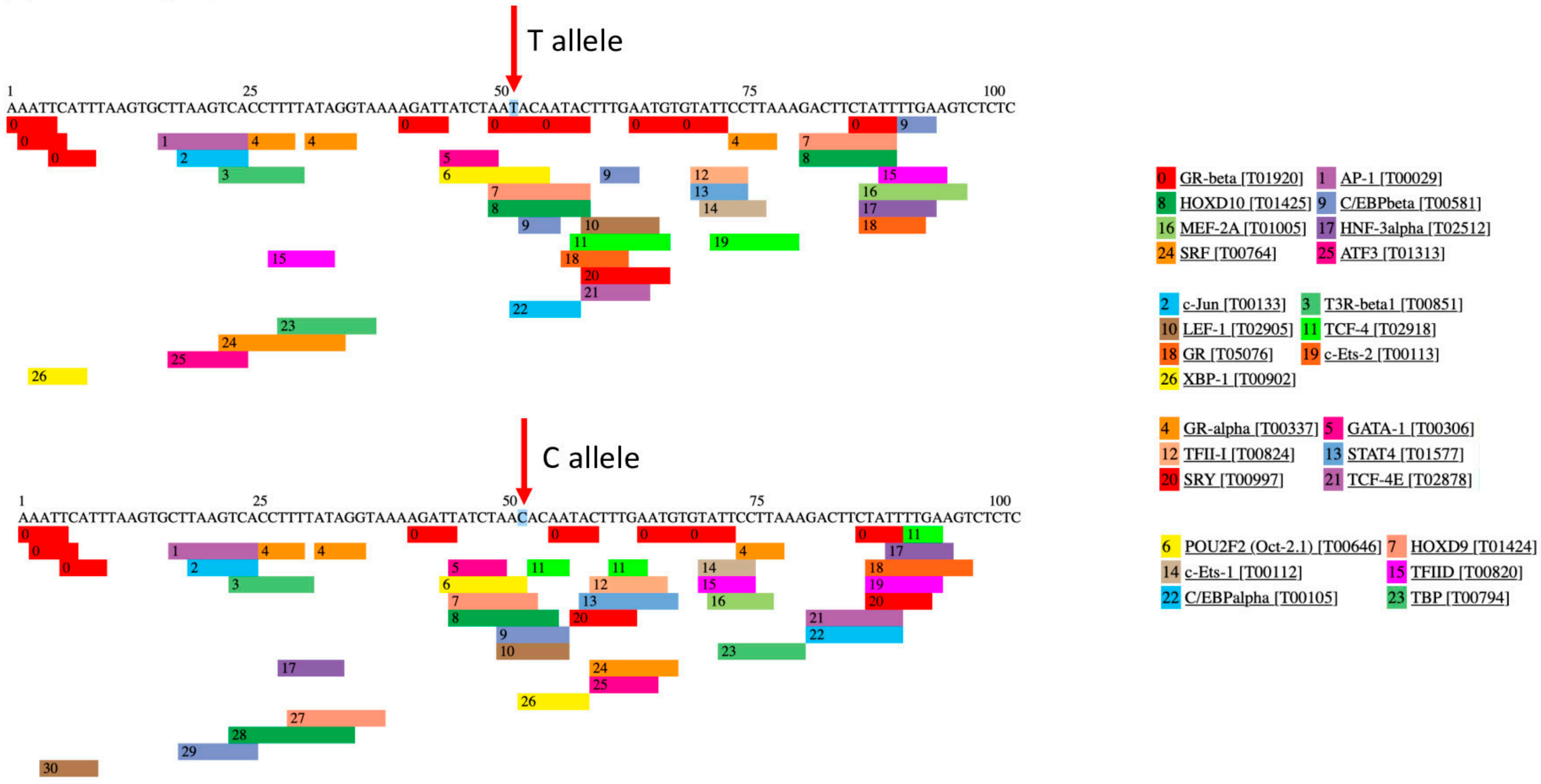

Supplementary Fig. 2. Transcription Factor Binding Sites Prediction for intronic SNP rs7116978 (PROMO, http://alggen.lsi.upc.es/cgi-bin/promo_v3/promo/promoinit.cgi?dirDB=TF_8.3). The red arrows indicate the two different alleles of the SNP. Transcription factors are represented in different colors

Supplementary Fig. 2. Transcription Factor Binding Sites Prediction for intronic SNP rs7116978 (PROMO, http://alggen.lsi.upc.es/cgi-bin/promo_v3/promo/promoinit.cgi?dirDB=TF_8.3). The red arrows indicate the two different alleles of the SNP. Transcription factors are represented in different color
